# Balloon dilation in sheath technique in transcatheter aortic valve replacement via narrow femoral artery: first-in-human early feasibility study

**DOI:** 10.3389/fmed.2025.1732507

**Published:** 2026-01-02

**Authors:** Xiaoqian Sun, Xiangjuan Liu, Luyao An, Liangyi Qie, Lianyue Ma, Xiao Meng, Huixia Lu, Guihua Yao, Mei Dong, Guipeng An

**Affiliations:** 1 State Key Laboratory for Innovation and Transformation of Luobing Theory, Key Laboratory of Cardiovascular Remodeling and Function Research of MOE, NHC, CAMS and Shandong Province, Department of Cardiology, Qilu Hospital of Shandong University, Jinan, China; 2 Department of Geriatric Medicine and Laboratory of Gerontology and Anti-Aging Research, Qilu Hospital of Shandong University, Jinan, China; 3 Department of Cardiology, Qilu Third Hospital of Shandong University, Qingdao, China

**Keywords:** early intervention, novel technique, precision medicine, safety, TAVR, transfemoral access

## Abstract

**Background:**

Severe aortic stenosis (AS) imposes sustained pressure overload on the left ventricle, leading to hypertrophy and myocardial fibrosis, which are key features of adverse cardiac remodeling. Timely transcatheter aortic valve replacement (TAVR) may reverse these processes. Transfemoral access remains the primary access method for TAVR, but the limited technique restricts early intervention in patients with narrow femoral arteries. This study aimed to evaluate the safety and feasibility of a novel balloon dilation in sheath (BinS) technique designed to facilitate transfemoral TAVR in such patients.

**Methods:**

This multicenter, prospective, and first-in-human early feasibility study included patients with severe AS and challenging femoral anatomy who underwent TAVR using the BinS technique between February 2023 and August 2024. The primary endpoint was the 30-day rate of major adverse events. The secondary endpoint was the rate of major adverse events at 6 months. The clinical endpoints included major post-procedural complications.

**Results:**

Fifteen patients (mean age 73.5 ± 7.3 years; 66.7% male) were treated successfully with the BinS technique via transfemoral access. There were no cases of all-cause mortality, stroke/transient ischemic attack, or severe major vascular access site complications defined by Valve Academic Research Consortium (VARC)-3 at both 30 days and 6 months. No myocardial infarction or paravalvular leakage was observed. 12 patients obtained VARC-3 technical success. Two patients experienced postprocedural arterial dissection, and two developed new-onset conduction abnormalities (left bundle branch block or permanent pacemaker implantation). At 30 days, left ventricular ejection fraction improved significantly from 50.8 ± 17.0% pre-procedure to 59.9 ± 14.6% post-procedure (*p* < 0.001).

**Conclusion:**

The BinS technique appears to be a safe and effective method for enlarging the narrow femoral lumens during TAVR. By broadening transfemoral TAVR eligibility, this approach may permit earlier hemodynamic unloading, thereby mitigating adverse cardiac remodeling and potentially improving long-term outcomes. Larger, longer-term studies are warranted to confirm these findings.

## Introduction

Aortic valve stenosis (AS) is a common heart disease in the elderly population, and its incidence gradually increases with age. According to statistics, about 2% of people over 65 years old have various degrees of AS, which has become the third largest cardiovascular disease after coronary heart disease and hypertension (1, 2). For elderly patients with severe symptomatic aortic valve stenosis, transcatheter aortic valve replacement (TAVR) has become a reliable and valid alternative treatment. Much of the data supporting TAVR showed durable benefits among patients at high, intermediate, or low surgical risk (3). PARTNER 3 (Safety and Effectiveness of the SAPIEN 3 Transcatheter Heart Valve in Low Risk Patients with Aortic Stenosis) demonstrated a reduction in the primary composite endpoint of all-cause death, stroke, and/or cardiac rehospitalization following TAVR, and these findings were sustained at 2 years (4). Meanwhile, health status through 5 years in intermediate-risk patients treated with a self-expanding TAVR prosthesis improved more rapidly than in patients treated with surgical aortic valve replacement (SAVR) (5). The primary consideration of TAVR operation is the femoral access. More than 90% of patients choose the femoral artery access (6). Besides, nonfemoral routes such as transapical access, transaortic access, transaxillary access, transcarotid access, transbrachiocephalic access, and transcaval access are used (7). In previous studies, transforaminal (TF) access was recommended as the optimal access route due to its minimal complications compared with nonfemoral routes (7, 8). However, approximately 15% of patients requiring TAVR have severe peripheral artery disease (1, 9), and it is difficult to perform TAVR via the lower extremity arterial access. With the iliofemoral minimum lumen diameter (MLD) less than 7.5 mm and other undesirable vascular conditions, alternative routing schemes are generally required (9). In addition, in the Asian population, the peripheral vascular system is more tortuous, and the femoral artery diameter is smaller. This may make the operation of TAVR via the femoral artery more difficult (10, 11). Our study pioneered a novel technique of Balloon Dilation in Sheath via the femoral artery to solve the difficult problem and the current study aimed to evaluate its safety and effectiveness during TAVR.

## Materials and methods

### Patient population

This was a multicenter and prospective study. A total of 15 patients who underwent TAVR via the femoral artery access utilizing the Balloon Dilation in Sheath (BinS) technique in Qilu Hospital of Shandong University, Zibo Central Hospital and Shengli Oilfield Central Hospital between February 2023 and August 2024 were included. All patients had symptomatic and severe aortic stenosis. After discussion by our interdisciplinary cardiac team, these patients were not candidates for open heart surgery, had a higher surgical risk, or had other clinical factors not reflected in traditional risk assessments, and chose a minimally invasive approach to treat severe AS. The preoperative evaluation suggested difficulty via the femoral artery access. After discussion, it was decided to use an expandable sheath and dilate the narrow femoral artery approach through an intrathecal balloon to perform TAVR. This study was approved by the Institutional Ethics Committee of Qilu Hospital of Shandong University and all recruited patients signed informed consent for the participant.

### TAVR procedure

Pre-procedure evaluation results were used to determine the degree of aortic valve stenosis and intervention risk stages and confirm the intervention time and benefits. Computed tomography (CT) and echocardiography were evaluated to clarify the structure of the aortic valve root, aortic valve regurgitation, aortic ring, approach selection, prosthetic valve selection, etc. Cases must be treated individually according to their clinical characteristics and the severity of heart failure. General or local anesthesia is used. Then, choose the main and secondary vascular accesses according to the condition of the blood vessels.

**Figure 1:**
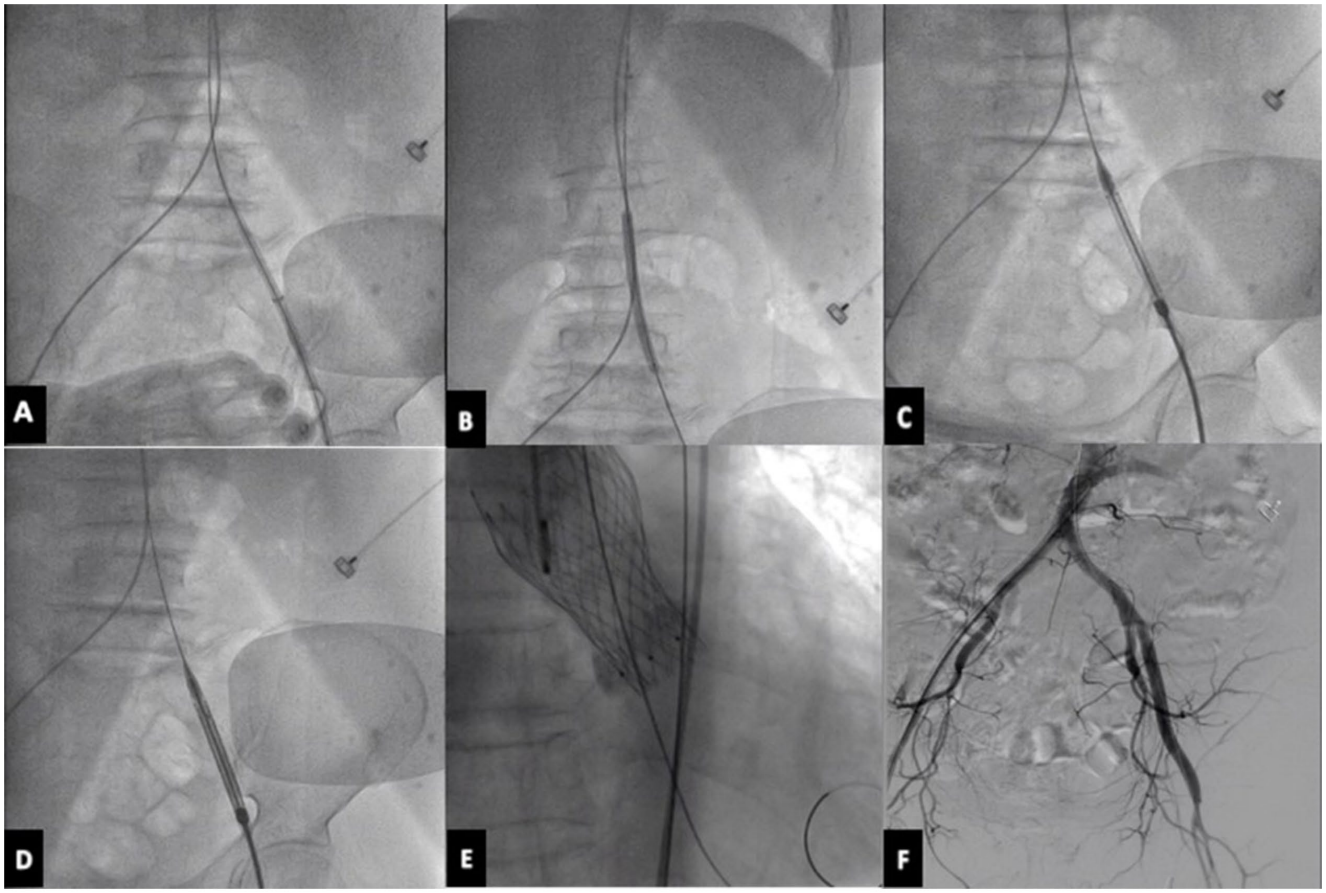
TAVR procedure. **(A)** Puncture the femoral artery with 16F expandable arterial sheath tube. **(B)** Use a balloon in the sheath to dilate the femoral artery stenosis. **(C)** Try to push the empty delivery system without a valve through. **(D)** Load and deliver the VENUS-A valve using the delivery system. **(E)** Release the prosthetic aortic valve. **(F)** Perform angiography on the iliofemoral artery to assess for any potential damage. TAVR, transcatheter aortic valve replacement.

The left or right femoral artery was used as the main approach, and the femoral artery was punctured with a 16F expandable arterial sheath tube and embedded 2 staplers in advance (Perclose Proglide). A 6 mm*10 cm or 6*8 cm balloon is used in the sheath to dilate the femoral artery stenosis and equally increase the vascular diameter. Intrathecal dilation can rely on the sheath to indirectly compress the blood vessels, so that the blood vessels are more evenly stressed, and the balloon dilation is safer and easier to obtain a larger lumen, reducing the risk of vascular dissection, vascular rupture, and vascular occlusion. Next, try to push the empty delivery system without a valve through, and then place the VENUS-A aortic valve. Angiography is performed to observe the position of the valve, whether the valve is released well, whether there is an obvious paravalvular fistula, and whether there is aortic valve regurgitation. Then the interventional valve delivery system is slowly withdrawn, meanwhile pushing the contrast agent, we should monitor this whole withdrawal procedure carefully under fluoroscopy. Finally, the iliofemoral artery is essential to be examined by angiography. If there is damage, the iliac or femoral artery should be quickly implanted with a stent along the guide wire (Figure 1).

### Data collection and endpoints

Baseline clinical and procedural data were prospectively collected from the electronic medical recording system. Follow-up data were collected during routine outpatient visits and via standard telephone interviews. Although the study involved multiple centers, echocardiography and CT measurements of patients were re-evaluated by the same dedicated imaging specialist from Qilu Hospital of Shandong University. Iliofemoral tortuosity (IFT) score measured by CT is associated with vascular access complications and bleeding complications and is a significant predictor of severe major vascular access complications. The calculation method has been described in a previous study (12).

The primary endpoint of the study was the 30-day rate of major adverse events (MAE), defined as the composite of all-cause mortality, stroke/transient ischemic attack (TIA), and main access site-related Valve Academic Research Consortium (VARC)-3 major vascular complications. The secondary endpoint was the rate of MAE at 6 months. The clinical endpoints included postprocedural access dissection, VARC-3 technical success, new-onset persistent left bundle branch block (NOP-LBBB) or permanent pacemaker implantation (PPI), myocardial infarction, and paravalvular leakage.

VARC-3 major vascular access site complications (13), defined as a vascular injury leading to death, life-threatening major bleeding, visceral ischemia, or neurological impairment, represent a significant proportion of the TAVR-related complications and constitute a significant burden for patients and healthcare facilities. VARC-3 technical success included (14): (1) freedom from death; (2) successful access, delivery of the device, and retrieval of the delivery system; (3) correct positioning of a single prosthetic heart valve into the proper anatomic location; and (4) freedom from surgery or intervention related to the device or a major vascular or access-related or cardiac structural complication. All endpoints were defined according to the VARC-3 definition (15).

### Statistical analysis

Continuous variables were presented as mean ± standard deviation if normally distributed or as median with interquartile range otherwise. Category variables were summarized as numbers and percentages. The paired Student’s *t*-test was employed to assess the difference between preprocedural and postprocedural data. All data analyses were performed using SPSS version 27.0 and Graphpad Prism version 9.5. A two-tailed test indicated that *p* < 0.05 was considered statistically significant.

## Results

### Baseline characteristics

The baseline characteristics of patients were shown in Table 1. In the overall population, the average age was 73.5 ± 7.3 years, with males representing 66.7% of the cohort. The prevalence of hypertension, diabetes mellitus, and coronary artery disease was 66.7, 26.7 and 86.7%, respectively. Before the operation of TAVR, four patients had atrial fibrillation, four had stroke/TIA, and 13.3% had cardiac bundle branch block. Regarding medication history, antiplatelet agents were prevalent, with 86.7% of patients on aspirin and 60.0% on clopidogrel, while 26.7% were receiving oral anticoagulation. Chronic use of heart failure and cardiovascular drugs included *β*-blockers (33.3%), ACEI/ARB/ARNI (26.7%), diuretics (26.7%) and digoxin (13.3%). Statin therapy was documented in 66.7% of patients, and calcium channel blockers in 20.0%. All patients suffered from severe aortic stenosis and one had moderate or severe aortic regurgitation. Mitral regurgitation was moderate/severe in 3 patients and moderate/severe tricuspid regurgitation occurred in 2 patients. The preprocedural left ventricular ejection fraction (LVEF) was 50.8 ± 17.0% and the preprocedural mean aortic valve gradient was 52.3 ± 13.8 mmHg. CT evaluation identified bicuspid valves in 46.7% of patients, and the mean aortic annulus area was 448.4 ± 81.4 mm^2^. On the main access side, the inner diameters of the common iliac artery, external iliac artery, and femoral artery were 5.4 ± 1.7 mm, 4.7 ± 1.4 mm, and 6.0 ± 1.3 mm, respectively. The median IFT score was 9.6 (IQR: 7.0–9.7). One patient with an IFT score of 30.17 experienced an external-iliac dissection post-procedure.

**Table 1:** Baseline characteristics.

Characteristic	Overall (*n* = 15)
Age, years	73.5 ± 7.3
Body mass index, kg/m^2^	22.8 ± 2.4
Male	10 (66.7)
NYHA functional class III or IV	8 (53.3)
Preprocedural NT-proBNP, pg./ml	2265.0 (951.7, 7555.0)
Preprocedural eGFR, mL/min/1.73 m^2^	79.3 ± 22.9
Comorbidities	
Hypertension	10 (66.7)
Diabetes mellitus	4 (26.7)
Prior atrial fibrillation	4 (26.7)
Prior bundle branch block	2 (13.3)
Prior stroke/TIA	4 (26.7)
Prior chronic renal insufficiency	3 (20.0)
Prior coronary artery disease	13 (86.7)
Medication history	
Aspirin	13 (86.7)
Clopidogrel	9 (60.0)
Oral anticoagulation	4 (26.7)
ACEI/ARB/ARNI	4 (26.7)
Beta blocker	5 (33.3)
Diuretics	4 (26.7)
Calcium channel blocker	3 (20.0)
Statin	10 (66.7)
Digoxin	2 (13.3)
Echocardiography	
Preprocedural left ventricular ejection fraction, %	50.8 ± 17.0
Preprocedural mean aortic valve gradient, mmHg	52.3 ± 13.8
Moderate/severe aortic regurgitation pre-TAVR	1 (6.7)
Moderate/severe mitral regurgitation pre-TAVR	3 (20.0)
Moderate/severe tricuspid regurgitation pre-TAVR	2 (13.3)
Computed tomography	
Bicuspid valve	7 (46.7)
Annulus area, mm^2^	448.4 ± 81.4
Left coronary height, mm	12.3 ± 2.1
Right coronary height, mm	16.1 ± 2.6
Low coronary height	2 (13.3)
Aortic angulation degree	46.8 ± 9.5
Common iliac artery inner diameter, mm	5.4 ± 1.7
External iliac artery inner diameter, mm	4.7 ± 1.4
Femoral artery inner diameter, mm	6.0 ± 1.3
IFT score	9.6 (7.0, 9.7)
Procedure	
General anesthesia	15 (100.0)
Valve size	
26 mm	10 (66.7)
29 mm	5 (33.3)
Perclose proglide	15 (100.0)

Values are mean ± SD, *n* (%), or median (IQR). Low coronary height was defined as either left or right coronary height ≤10 mm. eGFR, estimated glomerular filtration rate; NYHA, New York Heart Association; NT-proBNP, N-terminal B-type natriuretic peptide; IFT score, iliofemoral tortuosity score; TIA, transient ischaemic attack; TAVR, transcatheter aortic valve replacement.

### Procedural data and endpoints

**Figure 2:**
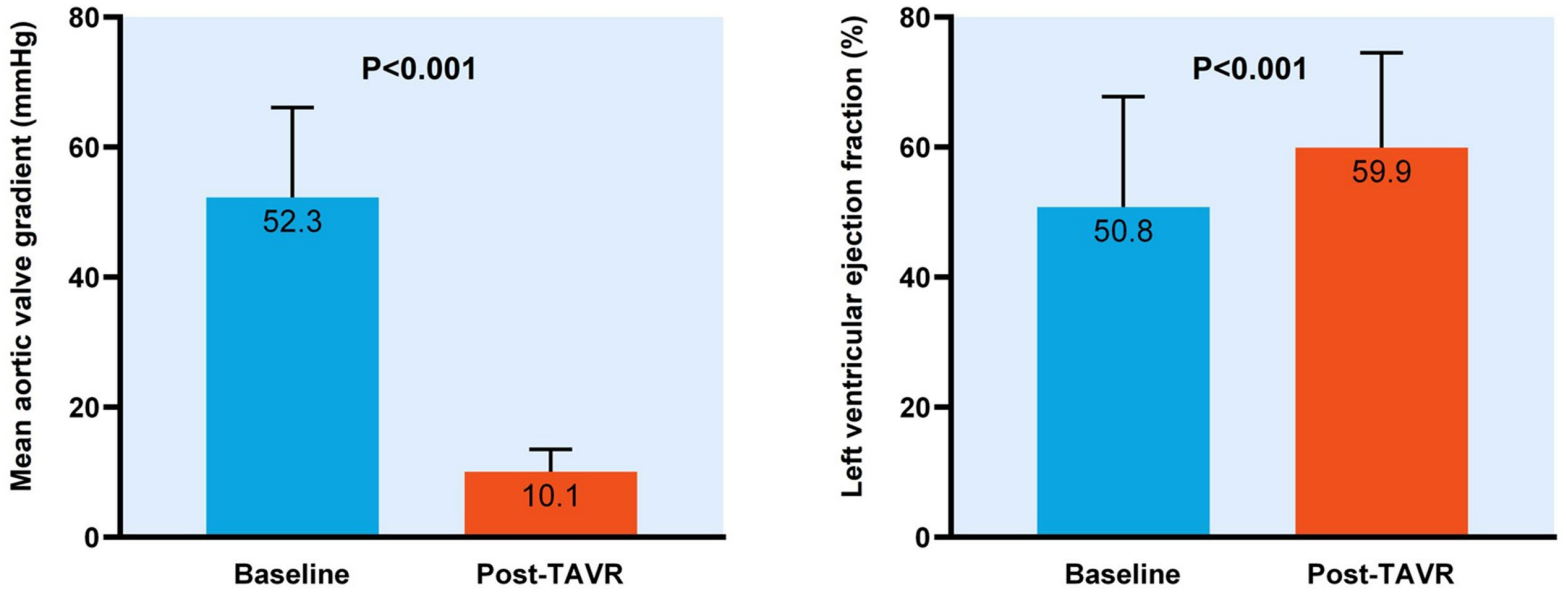
Change in mean aortic valve gradient and left ventricular eject fraction with transcatheter aortic valve replacement.

All procedures were performed general anesthesia. Most patients (66.7%) received 26 mm VENUS-A aortic valve implantation, and in the overall cohort, a perclose proglide was chosen. The artificial aortic valve was successfully placed after balloon dilation in the expandable sheath in all patients. Significant reduction of the mean aortic valve gradient (52.3 ± 13.8 mmHg vs. 10.1 ± 3.5 mmHg, *p* < 0.001, Figure 2) occurred with TAVR at 30-day follow-up, along with an increase in left ventricular ejection fraction (50.8 ± 17.0% vs. 59.9 ± 14.6%, *p* < 0.001, Figure 2). No all-cause mortality, stroke or TIA occurred after TAVR.

**Figure 3:**
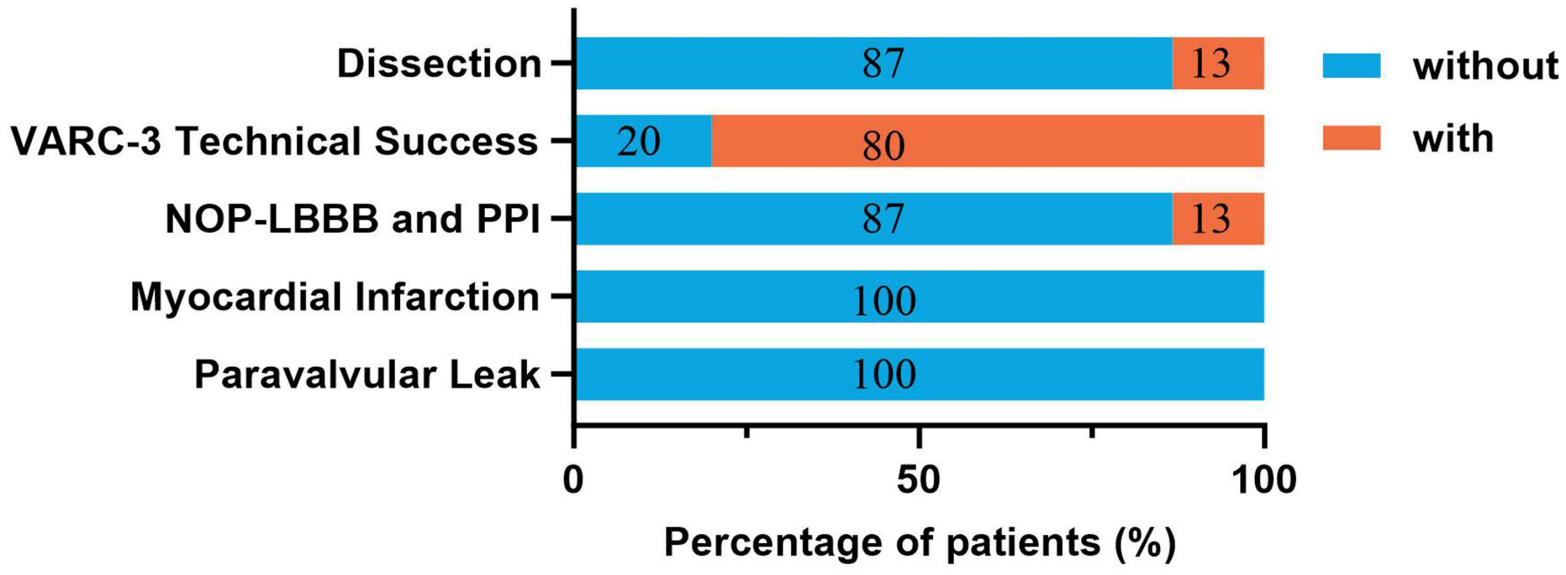
Clinical endpoints of the patients after TAVR. Values symbolize the percentage of patients who occurred or not occurred clinical endpoints after TAVR. NOP-LBBB, new-onset persistent left bundle branch block; PPI, permanent pacemaker implantation; VARC, Valve Academic Research Consortium; TAVR, transcatheter aortic valve replacement.

According to the primary and secondary endpoints, no MAE occurred at 30 days and 6 months follow-up (Table 2). Data regarding the clinical endpoints after the TAVR procedure were available for all patients (Figure 3). Two cases of external iliac artery dissection on the main access were observed after withdrawing the valve delivery system, which was successfully treated with stent implantation. One patient experienced bleeding from the main femoral access upon removal of the delivery system, which was resolved after placing a vascular closure device. The overall VARC-3 technical success rate was 80%. One new-onset persistent left bundle branch block and one permanent pacemaker implantation occurred. Notably, no post-procedural myocardial infarction or paravalvular leakage occurred.

**Table 2:** Primary and secondary endpoints.

Endpoint	Overall (*n* = 15)
Primary endpoint	0 (0)
All-cause mortality at 30 days	0 (0)
Stroke/TIA at 30 days	0 (0)
VARC-3 major vascular complications at 30 days	0 (0)
Secondary endpoint	0 (0)
All-cause mortality at 6 months	0 (0)
Stroke/TIA at 6 months	0 (0)
VARC-3 major vascular complications at 6 months	0 (0)

Values are *n* (%). TIA, transient ischemic attack; VARC, Valve Academic Research Consortium.

## Discussion

In this study, we described an expandable sheath used to improve transfemoral access when the condition of the femoral artery was poor. All procedures achieved technical success with a low incidence of major vascular access site complications. In addition, follow-up echocardiography demonstrated a modest but consistent improvement in LVEF, reflecting early recovery of systolic function following TAVR.

When transfemoral artery access is suboptimal, the procedural strategy should be considered in a comprehensive and individualized manner. Alternative access routes or adjunctive techniques may be employed when necessary. The carotid artery has become the second most used vascular access. Its principal advantage lies in its relatively straight anatomical course, which facilitates device manipulation, while the disadvantage is that it carries the potential risk of cerebral hypoperfusion, requires a surgical incision, and cannot be performed entirely percutaneously. In addition, several peripheral vascular intervention techniques may be adopted to optimize femoral access, including large sheath pre-dilation, selection of a small-sized large sheath, peripheral balloon pre-dilation technology, presetting technology of coated stent, intravascular lithotripsy and non-sheath technology (16–18). These strategies generally provide superior outcomes compared with non-femoral access routes during TAVR. However, the combination of TAVR and peripheral intervention may increase the risk of adverse events (19). For example, the pressure of the balloon should not be too large during the operation of percutaneous intravascular balloon dilation, because excessive balloon inflation may lead to arterial dissection or rupture. Balloon expansion after implantation of the covered stent is safer and easier to obtain a larger lumen, but attention should be paid to whether the covered stent is displaced when conveying the large sheath. Consequently, there remains a clear need to develop novel techniques that can overcome the challenges associated with transfemoral access and further refine the safety and efficacy of TAVR via the femoral access.

To solve the problem of femoral artery stenosis and difficult access, we pioneered the technique of balloon dilation in a sheath and opening an access route to assist TAVR. The BinS technique refers to those who underwent TAVR through the narrow femoral artery approach. The expandable 16F sheath is inserted into the vascular lumen, and the balloon is used to dilate in the sheath, increasing the vascular tube diameter, and assists the delivery of the TAVR device and the accomplishment of procedure. At present, the transfemoral valve delivery system listed in China is 18F or larger, and the narrowest diameter of the femoral artery is generally required to be above 7.5 mm. The use of small sheath sizes (14F or 16F) and expandable sheath tubes expands the application of the transfemoral approach to TAVR and significantly reduces the incidence of vascular complications. By performing balloon dilation within the sheath, the vessel wall is subjected to uniform, indirect radial force rather than direct balloon contact. This mechanism allows for safe luminal expansion while minimizing the risk of vessel dissection or rupture. It is crucial to perform angiography of the iliofemoral artery during sheath withdrawal. If damage occurs, a coated stent can be rapidly implanted to restore vessel integrity.

All fifteen patients in this study underwent TAVR using BinS, which was associated with a high level of procedural safety. Post-procedure vascular access complications have a significant impact on the prognosis of TAVR patients, which are closely related not only to prolonged hospitalization, reduced quality of life and increased mortality, but also to bleeding, wound infection and renal impairment, all of which contribute to higher procedural costs (20, 21). With the introduction of a new generation of TAVR device, the incidence of vascular complications was reduced. However, the incidence of life-threatening bleeding and major vascular complications were 3.9 and 4.5%, respectively, indicating that access site complications are still a major problem that needs to be addressed (22). According to the diagnostic criteria for postoperative vascular complications proposed by VARC-3, only one patient in the present study experienced a severe access-related bleeding event, and two cases of external iliac dissection occurred. Both were successfully treated with stent implantation, and subsequent angiography confirmed restoration of vessel patency without residual stenosis and dissection. When compared with alternative accesses, which are associated with higher complication rates and procedural complexity, the BinS technique provided a practical and safer means of achieving transfemoral TAVR in anatomically challenging patients.

In addition, the baseline medication history of our cohort is important for interpreting the vascular safety of the BinS technique. Most patients were receiving antiplatelet or anticoagulant therapy before TAVR, a pattern consistent with prior registries and technical reports (7, 17). Moreover, Brouwer et al. demonstrated that dual antiplatelet therapy increases periprocedural bleeding compared with aspirin alone, highlighting the sensitivity of large-bore access to intensified antithrombotic therapy (23). Despite the relatively high rate of antiplatelet or anticoagulant use in our study, the overall VARC-3 technical success rate was 80% and no VARC-3 major bleeding complications were observed at 30 days and 6 months. These findings suggested that the controlled radial force generated by balloon dilation within the expandable sheath might mitigate vessel trauma and reduce bleeding risk, even in patients receiving intensified antithrombotic therapy. Taken together, the medication history of our cohort further supported the procedural safety and feasibility of the BinS technique in patients undergoing transfemoral TAVR with challenging iliofemoral anatomy.

Regarding clinical outcomes, a small proportion of patients in this study developed new conduction abnormalities (NOP-LBBB or PPI) after the procedure. Given the pre-existing conduction disturbances and anatomical factors, the use of the BinS technique remained justified and did not appear to offset the overall procedural or functional benefits observed (24).

Importantly, post-procedural echocardiographic assessment demonstrated a significant improvement in LVEF and the limited use of ACEI/ARB/ARNI and *β*-blockers suggested that medical therapy played little role in cardiac recovery, further indicating that the improvement in LVEF might be driven by the hemodynamic benefits of timely valve intervention. In severe aortic stenosis, the left ventricle initially adapts to maintain cardiac output and normalize wall stress through hypertrophy. However, this macroscopic compensation is underpinned by pathological microscopic changes. Chronic pressure overload triggers mechanotransduction pathways and neurohormonal activation, initiating molecular cascades that drive the differentiation of fibroblasts into myofibroblasts (25, 26). This results in reactive interstitial fibrosis and focal replacement scarring, alongside cardiomyocyte hypertrophy, which collectively compromise myocardial mechanics (27). Consequently, TAVR allows for the immediate relief of this chronic pressure overload and initiates favorable left ventricular reverse remodeling following restoration of normal aortic valve hemodynamics (28–30). In this context, the BinS technique may ensure that these physiological benefits of hemodynamic unloading are extended to patients who might otherwise experience procedural delays or require higher-risk non-femoral access. However, it is important to acknowledge that our study is limited by its small sample size and the absence of longitudinal imaging markers for structural remodeling, such as cardiac magnetic resonance. Therefore, the observation regarding myocardial recovery should be considered preliminary and warrants validation in larger cohorts with long-term tissue characterization.

## Limitations

The following limitations of the study should be acknowledged. First, as a small-scale descriptive observational study without independent event adjudication, it is susceptible to inherent selection and reporting biases. Due to limited statistical power, no further stratified analysis was performed. Second, although the study utilized a multicenter design, the majority of data was derived from a single high-volume center. This imbalance may introduce institutional bias and limited generalizability. Third, the follow-ups were performed at 1 and 6 months, which were relatively short. This duration might preclude the evaluation of mid-to-long-term outcomes, such as valve durability, late structural deterioration, or sustained myocardial functional recovery. An extended follow-up incorporating imaging and functional evaluations would be essential to elucidate the long-term prognostic value. Therefore, larger, prospective, and multicenter studies with extended follow-up periods are essential to further validate the safety, efficacy, and long-term prognostic value of the BinS technique in patients with severe aortic stenosis with narrow femoral artery access.

## Conclusion

The current study demonstrated that the BinS technique might be a safe and effective approach, which might help to expand the indications of TAVR in patients with severe femoral artery stenosis. This method enables earlier intervention and effective hemodynamic unloading. These benefits may in turn promote favorable reverse cardiac remodeling and improved clinical recovery.

## Data Availability

The data set cannot be made publicly available because informed consent from participants did not cover public deposition of data. Requests to access the datasets should be directed to meidong_sdu@163.com.
